# Freckles

**Published:** 1883-09

**Authors:** 


### FRECKLES.

Freckles can be removed, according to Dr. J. V. Shoemaker, by the careful application of a little ointment of the oleate of copper at bed time. He makes the ointment by dissolving the oleate of copper in sufficient oleo-palmitic acid to make a mass.

